# Cross-feeding drives degradation of phthalate ester plasticizers in a bacterial consortium

**DOI:** 10.3389/fmicb.2025.1757196

**Published:** 2026-03-18

**Authors:** Simone Bertoldi, Simon Klaes, Stefanie Claus, Alex Marsans, Hermann J. Heipieper, Christian Eberlein

**Affiliations:** 1 Department of Molecular Environmental Biotechnology, Helmholtz Centre for Environmental Research – UFZ, Leipzig, Germany; 2 Department of Applied Microbial Ecology, Helmholtz Centre for Environmental Research– UFZ, Leipzig, Germany

**Keywords:** aromatics degradation, biorecycling, plastic, plasticizers, *Pseudomonas*

## Abstract

Reports of plastic pollution across diverse ecosystems continue to emphasize the environmental risks associated with the increasing consumption of synthetic polymers. Plastics frequently contain additives such as phthalic acid esters, which are extensively employed as plasticizers to enhance flexibility in plastic materials and as constituents of numerous consumer products. These compounds are not chemically bound to polymers, allowing them to leach into the environment and have been implicated as potential endocrine disruptors in animals. In the present study, the bacterial degradation of selected phthalate esters was examined, with diethyl phthalate (DEP) utilized as a model compound. A bacterial consortium capable of degrading DEP was enriched from a biofilm of a polyurethane tubing. The consortium was capable to mineralize DEP as the sole carbon and energy source at concentrations of up to 4 mM, whereas concentrations above 6 mM inhibited its activity due to DEP toxicity. This degradation was only possible by the whole consortium and not by single isolates. The degradation of DEP as well as the timely occurrence of monoethyl phthalate as degradation intermediate was confirmed by UPLC analysis. Metagenomic sequencing identified the consortium as comprising a *Microbacterium* sp. strain and two *Pseudomonas* spp. Metaproteomic analyses of the consortium, performed under varying time points and carbon sources and integrated with complementary growth experiments, facilitated the reconstruction of the degradation pathway and the identification of putative enzymes involved in DEP metabolism. *Microbacterium* sp. DEP1M initiated the degradation by hydrolysis of DEP into ethanol and monoethyl phthalate, which is then taken up by the cells and further metabolized to ethanol and phthalate. The latter is subsequently oxidized by a dioxygenase and further transformed to the central intermediate 3,4-dihydroxybenzoic acid (protocatechuate). Protocatechuate is then exclusively degraded via the *ortho* cleavage pathway. Notably, the distribution of enzymatic functions among different community members strongly supports the occurrence of microbial cross-feeding, indicating that DEP mineralization is a cooperative process within the consortium.

## 1 Introduction

Plastic degradation is a complex process influenced by several limiting factors. Physicochemical properties like crystallinity, hydrophobicity, and glass transition temperature often limit the possibilities for enzymatical attacks. The physicochemical properties of polymers can often be modified through the use of additives. Additives represent a major component in plastic production, accounting for about 55% of all chemicals incorporated into plastic (Monclús et al., 2025). Among additives, plasticizers play a particularly crucial role, as they significantly influence the mechanical and functional properties of plastics improving durability and flexibility. Plasticizers constitute an important segment of the plastic additives market and are also increasingly employed for purposes beyond traditional plastic applications (Wiesinger et al., 2021). Phthalic acid esters (PAEs), the most common plasticizers, are a group of ester compounds derived from phthalic acid (benzene-1,2-dicarboxylic acid), where two alkyl or aryl groups are esterified to the two carboxyl groups of phthalic acid.

Phthalic acid esters are considered harmful to health (Lange et al., 2022; European Human Biomonitoring Initiative, 2025). Because they are not chemically bound to polymers but incorporated through weak physical interactions (e.g., van der Waals forces and hydrogen bonding), they gradually leach from plastics into the surrounding environment (Thompson et al., 2009; Fauvelle et al., 2021). Reflecting these risks, their use is strictly regulated, particularly within the EU (European Chemicals Agency, 2021). Under REACH Annex XVII, four *ortho*-phthalates (DEHP: Di(2-ethylhexyl) phthalate, DBP: Dibutyl phthalate, BBP: Benzyl butyl phthalate, and DIBP: Diisobutyl phthalate) are restricted in consumer articles in the EU at concentrations of ≥0.1%. Because PAEs can migrate throughout the entire life cycle of plastics, they pose challenges to recycling by potentially compromising material quality and safety (Wiesinger et al., 2021; Carney Almroth et al., 2025). Their environmental release is well documented; for example, wastewater treatment plants act as major reservoirs, with sludge samples frequently showing high concentrations—especially of DEHP and DBP (Wang et al., 2022; Zhu et al., 2019). Whereas the use of the aforementioned PAEs is partially restricted, diethyl phthalate (DEP) is still widely used in cosmetics, fragrances, and various industrial applications, including plasticizers and aerosol sprays.

Several bacterial species were isolated able to grow on PAEs (Ren et al., 2018) from marine (Wright et al., 2020), terrestrial (Shariati et al., 2022) and freshwater (Lu et al., 2020). In most cases, like *Rhodococcus jostii* RHA1, single strains can only grow on monoalkyl PAE but not on the corresponding dialkyl PAE (Hara et al., 2010). An ATP-binding cassette (ABC) transporter encoded by *patDABC* is required for the uptake monoalkyl PAEs in *Rhodococcus jostii* RHA1 (Hara et al., 2010). Previous studies showed PAE degradation in microbial consortia and examined the biochemical cooperation between bacterial strains, with a focus on the hydrolysis of the ester bonds (Shariati et al., 2021; Lu et al., 2020). However, microbial cross-feeding throughout the complete mineralization process has not been investigated.

Therefore, we carried out a screening for DEP degrading bacteria that resulted in the isolation of a very stable bacterial consortium from a biofilm scraping of a polyurethane tubing. Using state-of-the-art metagenomic and metaproteomic techniques, a degradation pathway for DEP was proposed with putative key enzymes being identified.

## 2 Materials and methods

### 2.1 Isolation of bacterial consortium and substrate spectrum

A sample from a biofilm scraped off from a polyurethane tubing of a bioreactor was taken and incubated in mineral media, as reported before (Hartmans et al., 1989), containing the following compounds (per liter demineralized water): 7 g Na_2_HPO_4_ × 2 H_2_O; 2.8 g KH_2_PO_4_; 0.5 g NaCl; 0.1 g NH_4_Cl; 0.1 g MgSO_4_ × 7 H_2_O; 10 mg FeSO_4_; 5 mg MnSO_4_; 6.4 mg ZnCl_2_; 1 mg CaCl_2_ × 6 H_2_O; 0.6 mg BaCl_2_; 0.36 mg CoSO_4_ × 7 H_2_O; 0.36 mg CuSO_4_ × 5 H_2_O; 6.5 mg H_3_BO_3_; 10 mg EDTA; 2 M HCl (37%). 2 mM or 4 mM DEP (Sigma-Aldrich) were used as the main source of carbon and energy. Additionally, yeast extract was supplemented to cultures with PAEs as main growth substrates at a concentration of 200 mg L^−1^ to promote and stabilize microbial growth. To obtain a stable DEP degrading culture at least four transfers were done. 4 mM DMP (dimethyl phthalate), 1.6 mM DBP (dibutyl phthalate), 1.8 mM DPP (dipropyl phthalate), protocatechuate, phenol (1–4 mM), catechol, and benzene triol, phthalic acid (100–300 mg ^−1^) ethanol (1% v/v) were tested if they serve as source of carbon and energy.

Cultivation was done in 25 ml shaking cultures in 100 ml flasks, with aluminum lids, at 30 °C at 150 rpm. Optical density was measured at a wavelength of 560 nm (Agilent Cary 100 UV–Vis Spectrophotometer). *Microbacterium ginsengisoli* (DSM No.: 18659, Type strain) was used as a reference strain for fatty acid methyl esterification analysis. It was grown in liquid Hartmann’s media and yeast extract as carbon source, or in medium 92 (see DSMZ database), at pH 7.0–7.2 with the following composition: Trypticase soy broth 30.0 g, yeast extract 3.0 g, agar 15.0 g, distilled water 1000.0 ml.

The specific growth rate (*μ*) was calculated at the exponential phase of growth. Bacterial biomass was estimated assuming an OD_560nm_ of 1.0 corresponds to about 310 mg L^−1^ dry weight (Neumann et al., 2006). The growth yield (Y) was calculated according to the formula (all concentrations are expressed in mg L^−1^):


$$\;Y=(\frac{\mathrm{dry}\;\text{weight}}{\mathrm{DEP}-\text{phthalate accumulated}})\times 100\;$$

### 2.2 Quantification of DEP and potential degradation products via UPLC-PAD

Phthalic acid, monoethyl phthalate and diethyl phthalate were quantified using ultra performance liquid chromatography coupled to a photodiode array detector (UPLC-PAD). For this purpose, 500 μL of cultures grown in different conditions were collected and stored at −20 °C before analysis. On the day of measurement, samples were thawed and centrifuged at 20,817 × g at 4 °C for 10 min (Eppendorf centrifuge 5424R, rotor type FA-45-24-14) to remove particulate biomass. UPLC analysis performed using an AquityTM UPLC-PAD system (Waters, Eschborn, Germany) equipped with an AquityTM UPLC BEH C_18_ column (2.1 × 50 mm; 1.7 μm particle size, Waters, Eschborn, Germany), as described before (Hofmann and Schlosser, 2016). Samples were analyzed in triplicates. Compounds were eluted isocratically at a flow of 0.5 mL min^−1^. The eluent A was water + 10% MeOH + 0.1% formic acid (pH 3); the eluent B was MeOH + 0.1% formic acid (pH 3). The gradient was as follows: 0–2 min, 90% A/10% B; 2–3 min, 80% A/20% B; 3–5 min, 60% A/40% B; 5–5.5 min, 50% A/50% B; 5.5–8 min, 0.1% A/99.9% B; 8–8.2 min, 0.1% A/99.9% B; 8.2–8.5, 90% A/10% B. The detection wavelength was set to 278 nm. Quantification was calibrated using external standards of protocatechuate at 0.156–5 mM and phthalate, monoethyl phthalate, diethyl phthalate, and dimethyl phthalate at 0.125–4 mM.

### 2.3 Membrane phospholipid fatty acid composition

For the phospholipid fatty acids (PLFA) extraction, bacterial cells were harvested from an overnight culture and centrifuged for 7 min at 6000 × g. The pellet was washed with 1.5 mL of 10 mM KNO3, centrifuged and PLFA were extracted as reported before (Bligh and Dyer, 1959). Methylation was achieved by addition of 0.6 mL of 20% boron trifluoride in methanol (Morrison and Smith, 1964). The identification and quantification of the fatty acid methyl esters (FAME) was done using gas chromatography coupled to a flame ionization detection (GC-FID). Analyses were performed on an Agilent Technologies system equipped with a 7683B Series autosampler. Separation was carried out using an Agilent J&W CP-Sil 88 capillary column (50 m length, 0.25 mm internal diameter, 0.20 μm film thickness). Helium was used as the carrier gas at a constant flow rate of 1.0 mL min^−1^. Injections were performed in splitless mode with an injection volume of 1 μL and an injector temperature of 240 °C. The FID operated at 270 °C inlet temperature. The oven temperature program was as follows: initial temperature of 40 °C (held for 2 min), increased to 220 °C at a rate of 8 °C min^−1^, and held isothermally at 220 °C for 5 min. The pressure program started at 186.15 kPa for 2 min, followed by a linear increase to 310.26 kPa at 5.65 kPa min^−1^, with an isobaric hold at 315.09 kPa for 15.55 min. FAME were identified by co-injection of authentic reference compounds obtained from Supelco (Bellefonte, PA).

Additionally, to the standards used to identify FAME on the GC-FID, Gas Chromatography–Mass Spectrometry (GC–MS) analyses were carried out on an Agilent Technologies system consisting of a 7,890 GC system coupled to a 5975C inert XL MSD with a triple-axis detector and equipped with a 7,693 autosampler. Separation was achieved using a BP 5 forte GC capillary column (SGE, Darmstadt; 30 m length, 0.25 mm internal diameter, 0.25 μm film thickness). Helium was employed as the carrier gas at a constant flow rate of 1.2 mL min^−1^, corresponding to an average linear velocity of 25.81 cm s^−1^. The system was operated under isobaric conditions at 110 kPa. Injections were performed in splitless mode with an injection volume of 1 μL and an inlet temperature of 280 °C. The oven temperature program was as follows: initial temperature of 50 °C (held for 1 min), ramped to 250 °C at 4 °C/min, then increased to 300 °C at 20 °C/min, followed by an isothermal hold at 300 °C for 10 min.

### 2.4 16S rRNA gene amplicon and metagenomic sequencing

For DNA extraction, 2 ml of the cell cultures grown on DEP, succinate, protocatechuate, or LB-agar, were centrifuged at 6000 × g 10 min in 2 mL Eppendorf tubes to obtain cell pellets. DNA was extracted from cell pellets using the Nucleospin Microbial DNA kit from Macherey Nagel (Düren, Germany) following the manufacturer’s instructions. The DNA concentration was measured before and after the purification by DeNovix DS-11 + spectrophotometer. Three technical replicates for metagenome and one replicate for 16 rRNA gene sequencing, of purified extracted DNA, were sent to BGI Tech Solutions (Hong Kong) for 16S rRNA gene amplicon and metagenomic sequencing. For 16S rRNA gene amplicon sequencing, the V3–V4 hypervariable region was amplified using primers 338F (ACTCCTACGGGAGGCAGCAG) and 806R (GGACTACHVGGGTWTCTAAT), before sequencing in the paired-end mode (300 bp reads) on the DNBSEQ-G400 platform. For metagenomic sequencing, DNA libraries with 300–400 bp inserts were prepared and sequenced on the same platform in paired-end mode with 150 bp reads.

### 2.5 16S rRNA gene amplicon sequencing data analysis

BGI processed the raw sequencing reads as follows: primer and adapter sequences were removed using cutadapt; reads with an average Phred quality score below 20 within a 30 bp sliding window were truncated, and any read shortened to less than 75% of its original length was discarded; reads containing ambiguous nucleotides or regions of low complexity (10 identical consecutive bases) were also removed. The filtered reads provided from BGI were processed using DADA2 (Callahan et al., 2016) on the Galaxy (Abueg et al., 2024) instance of the Helmholtz Centre for Environmental Research – UFZ. Read quality profile were inspected with “dada2: plotQualityProfile.” Then, forward and reversed reads were filtered and trimmed with “dada2: filter and trim” and, afterwards, read quality profile was inspected again. The error rates in the amplicon dataset were estimated with “dada2: learnErrors.” The inference algorithm is applied to the sequence data with “dada2: dada” to pool the sequences. The pair forward and reversed reads are merged with “dada2: mergePairs.” The amplicon sequence variant (ASV) table was constructed with “dada2: makeSequenceTable.” Chimeras were removed with “dada2: removeBimeeraDenovo” and the final number of reads after the processing was assessed with “dada2: sequence counts.” The taxonomy was assigned to the sequence variants with “dada2: assignTaxonomy and addSpecies” using both databases GreenGenes2 (2024.09) and Silva 138.2 for comparison. A phyloseq object was created with “Create phyloseq object from dada2.” The file was exported and further processed in R (4.5.1) to plot the community compositions in different growth conditions using Ggplot. The full R code is available on GitLab (Link at the R codes-Simone Bertoldi / R_codes_publication · GitLab). The relative abundances of the strains were further grouped in *Pseudomonas fluorescens* group species and *Pseudomonas putida* group species and the bar plot was created according to the groups.

### 2.6 Metagenomic raw data processing

Raw metagenomic reads were quality-filtered by BGI to remove low-quality reads, adapter sequences, and reads with ambiguous bases using SOAPnuke (Chen et al., 2018) with the following parameters: “-n 0.001 -l 20 -q 0.5 --adaMis 3 --minReadLen 150.” Filtered reads were then assembled in a two-step strategy implemented by metaWRAP (Uritskiy et al., 2018): metaSPAdes (Nurk et al., 2017) v4.0 was first used to generate contigs ≥1.5 kb, the remaining reads were assembled with MEGAHIT (Li et al., 2015) v1.2.9 retaining contigs ≥1 kb, and subsequently contigs from both assemblies were combined. The combined contigs were binned using CONCOCT (Alneberg et al., 2014) v.1.1.0, MaxBin (Wu et al., 2016) v2.2.4, and MetaBAT (Kang et al., 2019) v2.12.1, and further refined and reassembled with metaWRAP v1.3.2. Bin quality was evaluated with CheckM (Parks et al., 2015) v1.0.18, and high-quality bins (completeness – 5 × contamination ≥50) were designated as metagenome-assembled genomes (MAGs) (Parks et al., 2017). MAGs were dereplicated at >99% ANI with dRep (Olm et al., 2017) v3.5.0, followed by taxonomic assignment with GTDB-Tk (Chaumeil et al., 2022) v2.4.0 (r220 database), and annotation with PGAP v6.10.

Table 1 shows an overview of the three strains of the bacterial consortium.

**Table 1:** Accession numbers and strain names of strains in the bacterial consortium.

Availability of metagenome derived genomes	Strain	Abbreviated as	Completeness [%]	Contamination [%]	Genome size [Mb]	Protein-coding genes
	*Pseudomonas putida* group sp.	DEP1T	98.30	1.06	5.59	5,184
https://doi.org/10.6084/m9.figshare.31042420	*Pseudomonas fluorescens* group sp.	DEP1C	99.93	0.14	6.27	5,656
	*Microbacterium* sp.	DEP1M	98.98	0.00	2.94	2,806

Completeness and contamination of the genomes was estimated via CheckM.

### 2.7 Metaproteomic analysis

Proteins were extracted from both cell pellet and supernatant of cultures grown for 48 h on DEP, succinate or protocatechuate. For cell pellet, frozen cell pellets from 2 mL culture, stored at −80 °C, using bead-beating, freeze–thaw, and sodium dodecyl sulfate treatment (SDS) followed by precipitation using trichloroacetic acid (TCA) as previously described (Tanca and Palomba, 2024; Zheng et al., 2024), but with some modifications. In brief, cell pellets were resuspended in 800 μL lysis buffer [1 M Tris–HCl pH 8, 4% (w/v) SDS, 10 mM dithiothreitol (DTT)]. Next, samples were subjected to bead-beating at 6.5 m s^−1^ for 1 min, 95 °C heat treatment at 500 RPM for 20 min, and freezing in liquid nitrogen subsequently. Then, samples were again subjected to bead-beating for 45 s (two times with 5 min break on ice) before freezing in liquid nitrogen, and heat treatment at 95 °C. The bead beating was repeated another time. After cell disruption, cell debris was removed by centrifugation at 14,000 × g for 10 min at room temperature, and the supernatant was collected. For supernatant, the previous protocol was modified as follows: 2 mL Aliquots of liquid cultures were collected and centrifuged (6,000 × g 10 min 4 °C). The supernatant was collected and further filtered via a 0.22 μm cellulose acetate filter. Proteins from cell pellet and supernatant were precipitated by adding trichloroacetic acid from a 100% (w/v) stock solution to the supernatant to a final concentration of 20% (v/v). Samples were mixed gently and incubated at −20 °C overnight. After centrifugation at 23,000 × g for 30 min at 4 °C, the supernatant was discarded. The resulting protein pellet was washed sequentially with 800 μL of 0.07% (v/v) *β*-mercaptoethanol and 1 mM phenylmethylsulphonyl fluoride in acetone, followed by 800 μL 80% (v/v) acetone in water, with centrifugation at 21,000 × g for 30 min at 4 °C after each wash. Residual acetone was removed by vacuum centrifugation. The washed pellet was resuspended in 30 μL of 100 mM ammonium bicarbonate (AMBIC, pH 8), supplemented with 5 μL of 2 ng μL^−1^ bovine serum albumin for quality control, and 35 μL of 10% (w/v) sodium deoxycholate (SDC) in water. Cysteine residues were reduced by adding DTT to a final concentration of 12 mM (incubated at 37 °C for 30 min at 400 RPM) and subsequently alkylated with 2-iodoacetamide at 40 mM (incubated at 20 °C in the dark for 45 min at 400 RPM). Samples were then diluted with 100 mM AMBIC buffer (pH 8) to a final SDC concentration of 1% (w/v) and digested with 0.63 μg sequencing-grade trypsin (Promega, Madison, WI, USA) overnight at 37 °C and 400 RPM. The digestion was terminated and SDC was precipitated by adding formic acid to a final concentration of 2.5% (v/v). To remove precipitates, the samples were sequentially centrifuged three times at 16,000 × g for 10 min, with the supernatant from each spin used for the next centrifugation. Peptides were desalted using Pierce C_18_ tips (Thermo Scientific, Waltham, MA, USA) following the manufacturer’s instructions, dried by vacuum centrifugation, and stored at −20 °C *prior* to mass spectrometry, peptides were resuspended in 30 μL of 0.1% (v/v) formic acid, and 6 μL was injected into the nLC-MS/MS system.

Peptides derived from cell pellets were analyzed by nLC-FAIMS-MS/MS on an Orbitrap mass spectrometer operated in data-dependent acquisition (DDA) mode. Instrument parameters were as described previously for in-solution digested samples (Klaes et al., 2023) with the following modifications: a dynamic exclusion time of 45 s was applied, and FAIMS Pro separation was enabled using compensation voltages (CVs) of −40 and −55 V with a cycle time of 1.5 s per CV. Peptides obtained from culture supernatants were analyzed by nLC-MS/MS on the same Orbitrap platform in DDA mode without FAIMS. Acquisition parameters were identical to those described previously (Klaes et al., 2023), except that a dynamic exclusion time of 45 s was used and precursor ions with charge states +2 to +4 were selected for fragmentation. Mass spectrometry raw data were processed using Proteome Discoverer v3.2. Database searching was performed using CHIMERYS (Frejno et al., 2025) against a metagenome-derived protein sequence database with 14,025 entries, created from the 13,646 protein-coding genes translated from the dereplicated MAGs and appended with 379 universal contaminants (Frankenfield et al., 2022). We allowed a fragment mass tolerance of ±0.1 Da, peptide lengths of 7–30 amino acids, and a maximum of two missed trypsin cleavages. Oxidation of methionine and carbamidomethylation of cysteine were set as dynamic and static modifications, respectively. The false discovery rate (FDR) was set to 1% at PSM, peptide, and protein levels. Label-free quantification was performed via the Minora Feature Detector based on precursor ion intensities. Protein abundances were normalized relative to the identified proteins of each individual MAG to assess changes in protein expression within a species, excluding proteins assigned to other MAGs. Abundance ratios between samples groups were calculated from the individual normalized protein abundances. Significance was tested using a *t*-test on the individual normalized protein abundances, with *p*-values corrected for multiple testing using the Benjamini-Hochberg (Benjamini and Hochberg, 1995) procedure. Proteins were considered as reliably identified target proteins if classified as high confidence, non-contaminant Master Proteins with at least two unique peptides identified. Reliably identified target proteins with |log2(abundance ratio)| > 1 and adjusted *p*-value < 0.05 were considered significantly differentially abundant.

### 2.8 Identification of enzymes putatively involved in diethyl phthalate degradation

To identify potential similarities with known enzymes, such as ester hydrolases or mono- and dioxygenases involved in aromatic compound degradation, the protein-coding genes translated from the MAGs were blasted against selected enzymes from the UniProtKB and the NCBI database using blastp (Altschul et al., 1997) or selected by their NCBI annotation/Enzyme Commission number (Schwengers et al., 2021).

Potential diethyl phthalate hydrolase candidates were identified by analyzing pellet and supernatant metaproteomes and selecting proteins showing significant differential production, using R (4.5.1) (Link at the R codes-Simone Bertoldi/R_codes_publication · GitLab). Proteins were considered as significantly differentially produced when flagged with |log2(abundance ratio)| > 1 and *p*-value < 0.05 in different conditions (DEP over time, DEP versus succinate, DEP versus protocatechuate). The localizations (cytoplasm, cytoplasmic membrane, periplasm, outer membrane, cell wall and surface, and extracellular space) of the proteins encoded by the MAGs were predicted using DeepLocPro1.0 (Moreno et al., 2024). Extracellular and outer membrane proteins were considered for further consideration as diethyl phthalate hydrolase candidates. The proteins were then manually selected based on the activity they catalyze (esterase activity). Peptidases and endonucleases were excluded from the candidates list.

For the identification of phthalate dioxygenase, phthalate dehydrogenase and phthalate decarboxylase candidates, Galaxy server (Galaxy-Galaxy Version 2.16.0 + galaxy0) was employed to perform BLASTp alignments (tool NCBI BLAST+ Blastp Search protein database with protein query sequences). The protein sequences encoded by the MAGs were used as a BLAST database and sequences selected from UniProt database were queried. Alignments with query coverage >80%, percentage identity >25% and E-value <1 × 10^−8^ were considered as potential candidates. The differential protein production of the potential candidates was further investigated. For candidates for a phthalate dioxygenase the following query sequences from Uniprot were used: Q05183, Q05182, Q68YB5, Q0RWD5, B7X5F0, A4ZXZ3, A0A1I4GN37, A0A1B1KG76, A0A1B5DCB8, A0A2Z5UDX3, A0A5E6TNB0, A0A0U3DCV9, A0A0F0L803, A0A0M2HQY3, A0A1Q9QZ31, Q3C1D5, Q3C1E0, Q3C1D4, Q3C1D2, P33164. For candidates for a phthalate dihydrodiol dehydrogenase the following query sequences were used: Q68YA3, UPI00085784EB, UPI00005D608A, UPI00004E5877. For candidates for a phthalate decarboxylase the following query sequences were used: Q0RWC9, Q9AGK2, Q93UV7, Q05185, Q59727. The enzymes involved in the *ortho*-cleavage pathway of protocatechuate were identified from literature and their presence was further investigated in metagenome and metaproteome. Proteins were considered as reliably present when flagged with high signal from MS/MS spectra in at least two of the three replicates of cells grown on DEP. Heatmaps of the differential production of enzymes involved in the *ortho*-cleavage pathway for protocatechuate degradation comparing different conditions (DEP versus succinate, DEP versus protocatechuate) were created using R Software (R 4.5.1). Link at the R codes-Simone Bertoldi/R_codes_publication · GitLab.

## 3 Results

### 3.1 Isolation and characterization of a microbial consortium growing on DEP

**Figure 1:**
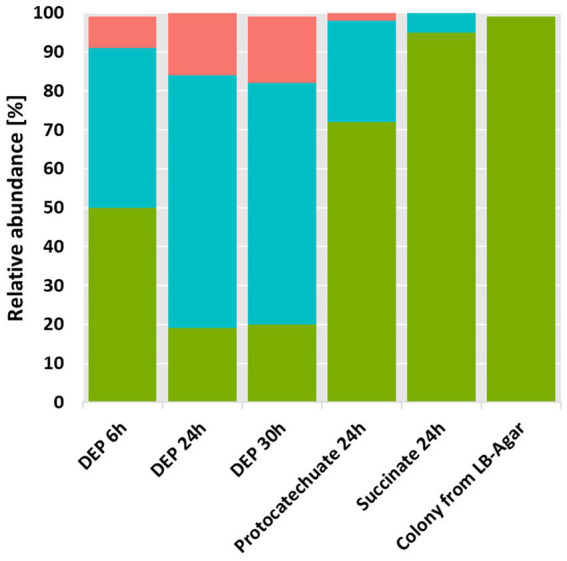
16S rRNA gene sequencing of the microbial consortium grown on 2 mM DEP (three different time points are shown), 5 mM protocatechuate, and 4 g/L succinate. The relative abundance for each strain is shown. In coral *Microbacterium* sp. DEP1M, in green *Pseudomonas putida* group, represented by DEP1T in DEP grown cells, in cyan *Pseudomonas fluorescens* represented by DEP1C in DEP grown cells.

The screening of samples collected from a biofilm of a polyurethane tubing resulted in the isolation of a microbial consortium degrading up to 4 mM DEP as the sole carbon and energy source. The metagenome as well as the 16S rRNA gene amplicon sequencing revealed that the microbial consortium degrading DEP consists of mainly three bacteria: one species of the *Pseudomonas putida* group (DEP1T), one of the *Pseudomonas fluorescens* group (DEP1C) and a *Microbacterium* sp. (DEP1M). The composition of the consortium changed when it was cultivated on other growth substrates such succinate or protocatechuate (Figure 1). During growth on DEP, strain DEP1C became dominant within the consortium. Isolated colonies from LB agar were identified as DEP1T and lose DEP-degrading capacity. The same strain is enriched when the culture is grown on protocatechuate and dominant when the consortium is grown on succinate. It is worth noting that 16S rRNA gene sequencing yielded different results at the species level depending on the database employed (Greengenes2 or SILVA 138.2). Similarly, metagenome sequencing also provided slightly different species-level assignments. Therefore, each strain was identified at the genus and group level, as reported in the literature (Girard et al., 2021; Gomila et al., 2015; Hesse et al., 2018).

### 3.2 Membrane phospholipid fatty acid (PLFA) analysis

GC–MS and GC-FID analysis confirmed the presence of unsaturated fatty acids (C16:1Δ9*trans*, C16:1Δ9*cis*, C18:1Δ11*trans*, and C18:1Δ11*cis*) that are very specific for *Pseudomonas* (Heipieper and De Bont, 1994) and the branched fatty acids C15:0*anteiso*, C16:0*iso*, and C17:0*anteiso*, which are very specific from Gram-positive bacteria such as *Microbacterium* (Kumari et al., 2013). Growing the consortium on different substrates (succinate, protocatechuate, DEP) resulted in significantly different PLFA patterns with a far higher abundance of branched fatty acids after growth with DEP (1Supplementary Figure S1). This strongly supports the shifts observed in the 16S rRNA gene sequencing data.

### 3.3 Growth yields, metabolites and substrate spectrum

**Figure 2:**
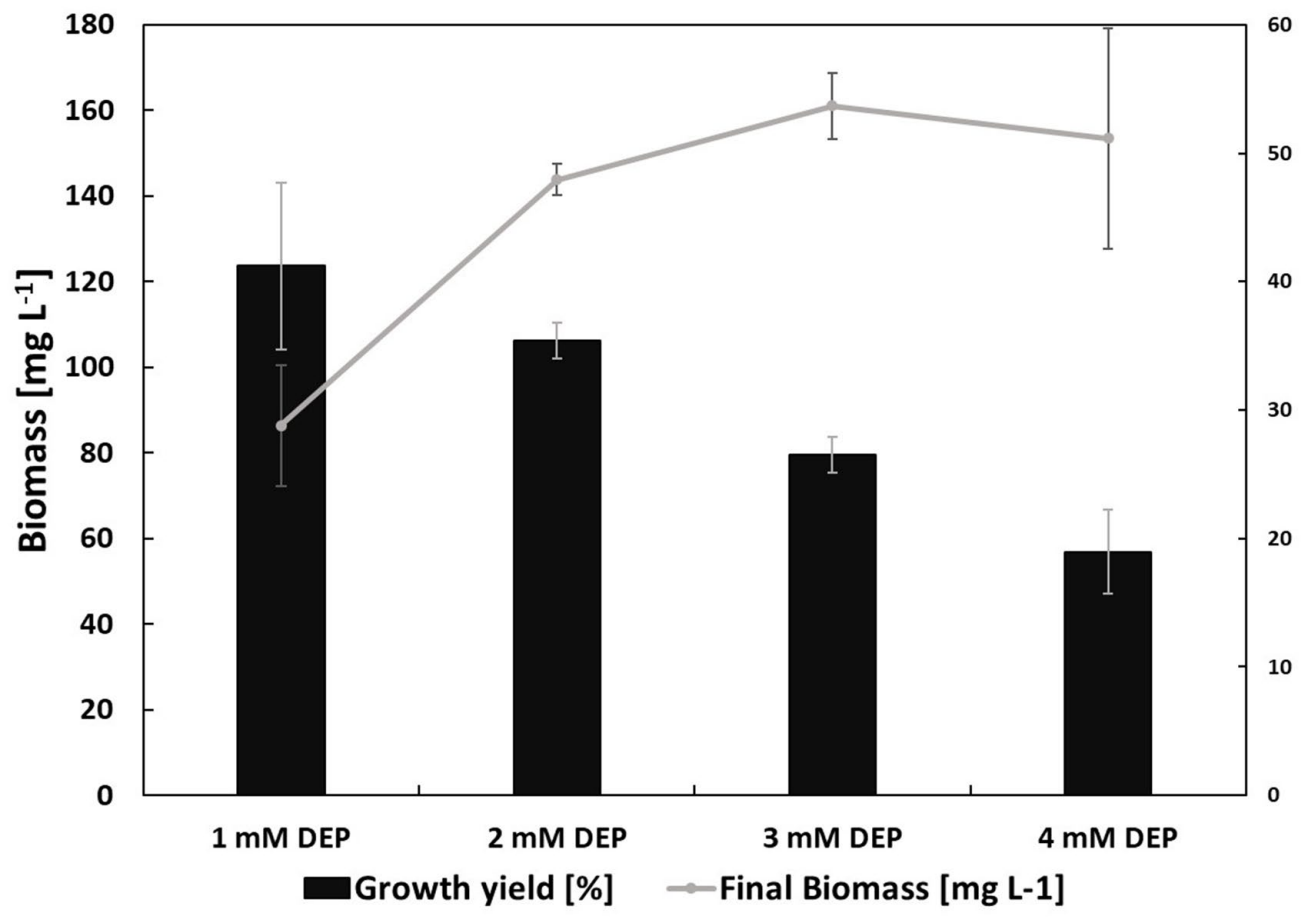
Bar plot showing the growth yields Y (black bars) and biomass formation (gray line) for the bacterial consortium on different concentrations of DEP at 22 h of growth. Specific growth rates: *μ* = 0.04 h^−1^ on 1 mM DEP and *μ* = 0.06 h^−1^ on 2, 3, and 4 mM DEP. Growth was completely inhibited at 6 mM. All calculations were based on the mean values of biological replicates (*n* = 3).

The microbial consortium was capable to grow on diethyl phthalate as carbon source in concentrations of up to 4 mM (888 mg L^−1^, Figure 2). The growth rate *μ* was about 0.04 h^−1^ for 1 mM DEP and increased up to 0.06 h^−1^ for all higher concentrations of DEP. However, the growth yield decreased from 41% in 1 mM DEP to 19% in 4 mM DEP cultures. The growth yield for the culture grown on the sole yeast extract was instead 16%. Cultures grown on 2 mM DEP (444 mg L^−1^) were chosen for further analyses.

**Figure 3:**
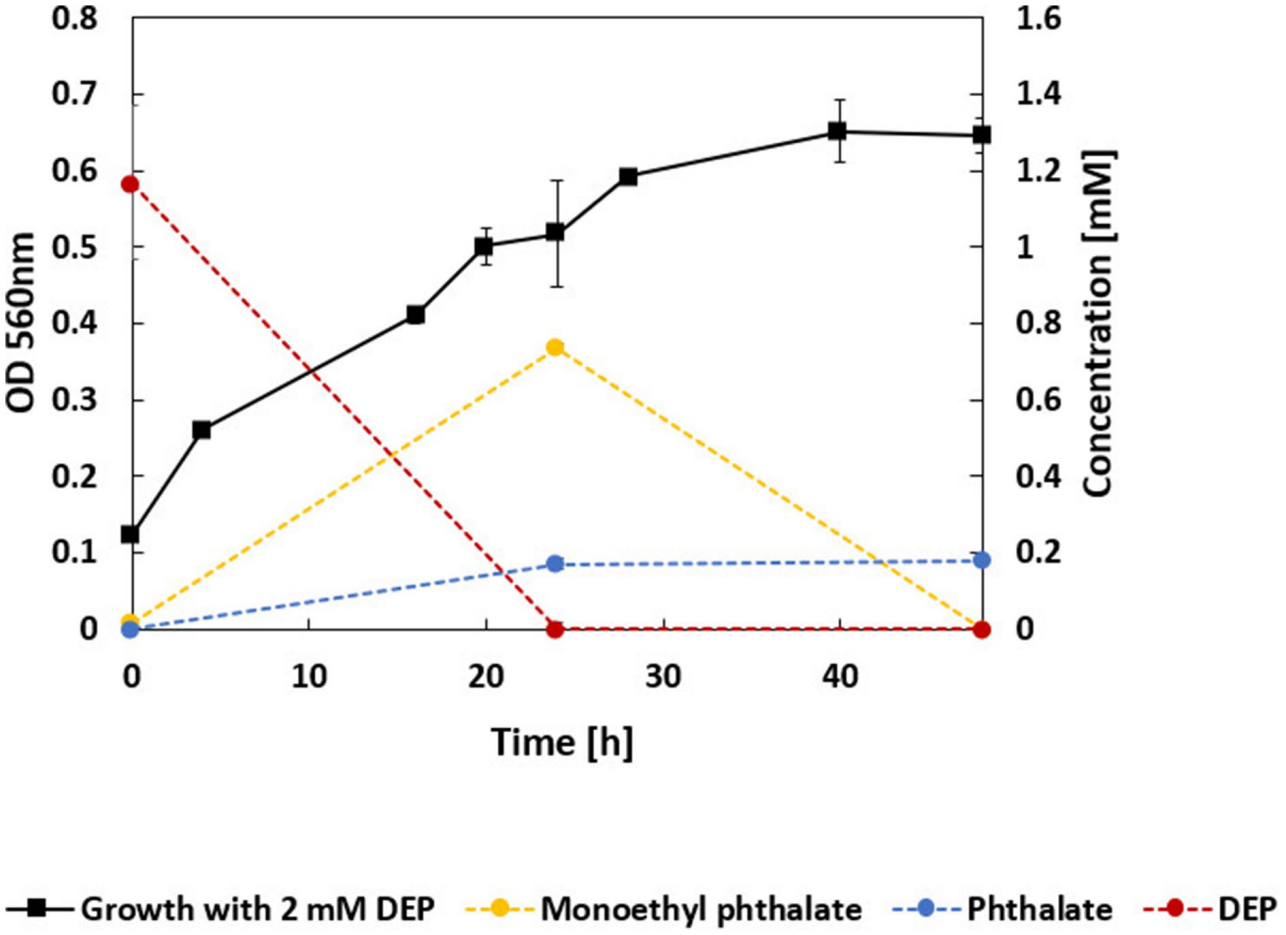
Growth curve and concentrations of DEP and its main degradation intermediates (DEP in red, monoethyl phthalate in yellow, phthalate (=salt of phthalic acid) in blue). 2 mM DEP was supplied as carbon and energy source. DEP concentrations were stable in sterile controls. Protocatechuate was not detected.

For growth on 2 mM DEP, an OD_560_ of approximately 0.65 was reached after 40 h of incubation (Figure 3). UPLC analyses showed that DEP was completely consumed after 24 h of growth. Concomitantly, monoethyl phthalate transiently accumulated in the culture supernatant, reaching a maximum at 24 h, but was no longer detectable after 48 h. In contrast, phthalate accumulation stabilized at around 0.2 mM.

A broad range of aromatic and non-aromatic compounds were tested as carbon sources to evaluate the metabolic versatility of the microbial consortium (Table 2). In addition to DEP, the consortium was able to grow on three other phthalate ester plasticizers with different alkyl chain lengths: dimethyl phthalate (388 mg L^−1^), dipropyl phthalate (444 mg L^−1^) and dibutyl phthalate (444 mg L^−1^). Ethanol and all tested aromatic compounds also supported growth, whereas phthalate itself did not serve as a carbon source for the consortium.

**Table 2:** Substrates tested as carbon and energy sources for the microbial consortium: 4.5 mM catechol, 4 mM phenol, 4 mM benzene triol, 4 mM dimethyl phthalate (DMP), 2 mM DEP, 1.6 mM dibutyl phthalate (DBP), 1.8 mM dipropyl phthalate (DPP), 3 mM phthalate, 10 mM 3,4-dihydroxybenzoic acid, 1% v/v ethanol.

Carbon source	Growth
Catechol	+
Phenol	+
Benzene triol	+
Ethanol	+
DMP	+
DEP	+
DBP	+
DPP	+
Phthalate	−
3,4-dihydroxybenzoic acid	+
Ethanol	+

### 3.4 Differential protein production (DEP versus succinate)

**Figure 4:**
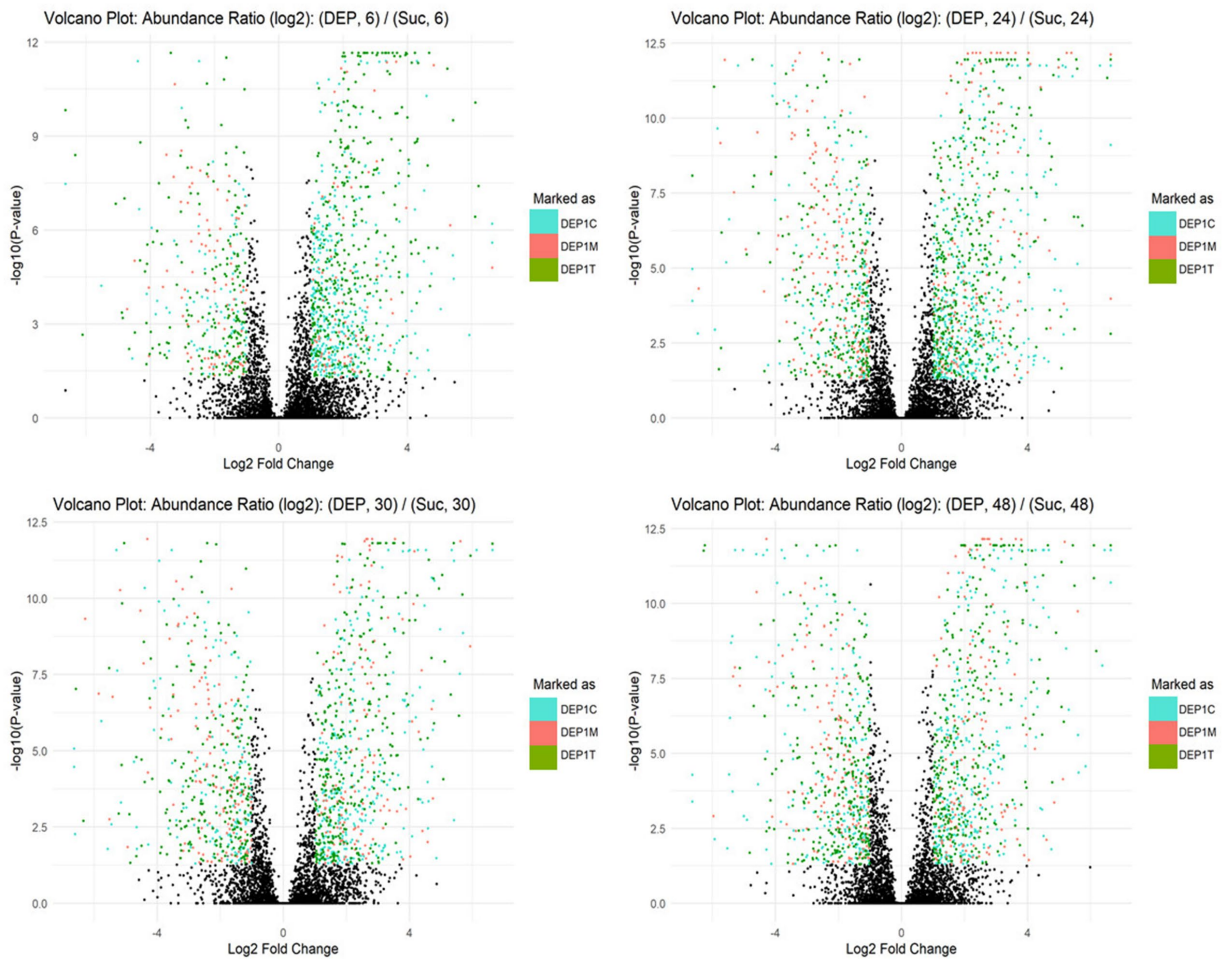
Volcano plots showing differential protein abundance in the metaproteome of the bacterial consortium grown on diethyl phthalate (DEP) compared to succinate at different incubation times (6, 24, 30, and 48 h). Each point represents a protein, with the *x*-axis indicating the log₂ fold change in abundance (DEP vs. succinate) and the *y*-axis showing the –log₁₀(*p*-value). Colored points correspond to proteins with –log₁₀(*p*-value) > 1.30 and log₂ fold change> 1, identified in each consortium member: DEP1C (cyan), DEP1M (pink), and DEP1T (green).

Metaproteomic analyses were conducted to investigate the metabolic activity of each strain when grown on DEP, protocatechuate, or the non-aromatic carbon source succinate, with the aim of identifying candidate enzymes involved in DEP degradation (Figure 4). The Volcano plots illustrate the differential protein abundance in the metaproteome of the bacterial consortium grown on DEP compared to succinate at different incubation times (6, 24, 30, and 48 h). Across all time points, several proteins were significantly upregulated in the DEP-grown cultures, indicating active metabolic adaptation to DEP as carbon source. The number and magnitude of differentially produced proteins increased over time.

### 3.5 Enzymes potentially involved in the degradation of DEP

The metagenome-resolved metaproteome analysis was integrated with the quantification of transformation products, to elucidate the putative DEP degradation pathway and to infer the potential roles of individual consortium members and their encoded enzymes involved in this process. A summary of the most likely enzymes for the DEP degradation pathway is shown in Table 3. Additional candidates and information can be retrieved from the Supplementary Tables S1–S3.

**Table 3:** Summary of the enzymes involved in the degradation of DEP in the microbial consortium.

Strain	Protein found in metaproteome of DEP grown cells	Accession	Potential role in DEP degradation	Upregulated DEP vs. succinate
DEP1M	Short-chain-length polyhydroxyalkanoate depolymerase	pgaptmp_001431	DEP hydrolase	Yes
DEP1C	Anthranilate 1,2-dioxygenase large subunit	pgaptmp_003809	Phthalate 3,4-dioxygenase	Yes
DEP1M	Aromatic ring- Rieske 2Fe-2S domain-containing protein	pgaptmp_002256	Phthalate 3,4-dioxygenase	Yes

Monoethyl phthalate was detected in the culture supernatant by UPLC analysis, confirming the extracellular hydrolysis of DEP. Several candidates for the hydrolytic cleavage of one ethanol residue of diethyl phthalate to monoethyl phthalate were identified to be highly overexpressed in the consortium. Interestingly, nearly all dialkyl hydrolases reported in the literature and examined in this study (Supplementary Table S4) were predicted by DeepLocPro 1.0 to be intracellular proteins. Only two alkyl hydrolases exhibited a predicted extracellular localization and were therefore selected for further analysis: dphBL1 (OM455495.1) and GoEst15 (MH513611.1). The only substantial sequence similarity detected was to a carboxylesterase from *Microbacterium* sp. DEP1M (pgaptmp_002689), with 98% query coverage and 34 and 38% sequence identity, respectively. However, metaproteomic analysis did not reveal any significant overproduction of these enzymes. The most probable extracellular enzyme, with a predicted localization probability of 0.999 according to DeepLocPro 1.0, is encoded in *Microbacterium* strain DEP1M (pgaptmp_001431). It is annotated as an extracellular short-chain-length polyhydroxyalkanoate depolymerase. It shares 99.3% sequence identity with a polyhydroxybutyrate depolymerase from *M. ginsengisoli* (UniProt A0A0F0LZZ6) and 37.9% with a homolog from *Ralstonia pickettii* (UniProt P12625), the latter of which has a verified enzymatic function (Saito et al., 1989). An additional potential hydrolase candidate (0.997 probability of extracellular location according to DeepLocPro 1.0) from DEP1M (pgaptmp_001484), was annotated as a cellulase family glycosyl hydrolase (Supplementary Table S1). However, this enzyme primarily catalyzes the hydrolysis of (1 → 4)-*β*-D-glucosidic linkages in polysaccharides such as cellulose and lichenin and is therefore unlikely to possess esterase activity (Souza and Kawaguti, 2021). Therefore, strain DEP1M is proposed to catalyze the initial hydrolytic cleavage of the first ester bond in DEP via a protein annotated as a polyhydroxyalkanoate depolymerase (pgaptmp_001431), which functions as the DEP hydrolase. This reaction could enable the release and subsequent uptake of the product, monoethyl phthalate, by other members of the consortium. The reaction intermediate, monoethyl phthalate, accumulated transiently in the culture medium (Figure 3). The second hydrolytic step, involving cleavage of the remaining ester bond, is readily catalyzed by an intracellular hydrolase and was not further investigated due to the presence of numerous candidate enzymes potentially capable of this reaction. The product of the two subsequent ester hydrolysis steps is phthalate. The phthalate accumulated in the supernatant (side product of DEP hydrolase activity) is distinct from intracellular phthalate potentially formed as an intermediate. The intracellularly formed phthalate could be a substrate for a phthalate dioxygenase, a common enzyme involved in phthalate degradation. An alignment of 21 sequences from UniProt annotated as phthalate 3,4-dioxygenase, phthalate 4,5-dioxygenase, or terephthalate 1,2-dioxygenase was used to identify potential phthalate dioxygenases in the bacterial consortium. Among the best alignments, only one potential phthalate dioxygenase (pgaptmp_003809) was present in strain DEP1C and was differentially overproduced in DEP compared to succinate or protocatechuate (Table 3 and Supplementary Table S2). Pgaptmp_003809 shows the highest similarity with A0A2Z5UDX3, a phthalate dioxygenase large subunit from *Pseudomonas* sp., with 95% query coverage and 33% sequence identity. The same sequence also displays 26% identity and 92% query coverage with Q3C1D5, a reviewed terephthalate 1,2-dioxygenase terminal oxygenase component subunit alpha from *Comamonas* sp. Pgaptmp_003809 was significantly overproduced in the presence of DEP, compared to succinate or protocatechuate (Table 3 and Supplementary Table S2). Moreover, pgaptmp_002256 of strain DEP1M, annotated as Rieske 2Fe-2S domain-containing protein, shows the highest similarity with A0A1I4GN37, a phthalate 3,4-dioxygenase *alpha* subunit from *Geodermatophilus ruber*, (95% query coverage, 79% sequence identity). The same protein also shows 100% query coverage and 74% sequence identity with A0A0U3DCV9, a phthalate dioxygenase large subunit from *Microbacterium* sp. J-1 (Table 3 and Supplementary Table S2). Supplementary Table S2 also shows candidate proteins for a subsequent reduction and decarboxylation steps. These candidates were identified in strains DEP1C and DEP1M. These reactions ultimately yield protocatechuate, a central intermediate of aerobic aromatic compound degradation.

Only the proteome of strain *Pseudomonas* DEP1C contained the complete *ortho*-cleavage pathway for protocatechuate (pgaptmp_003674, 003675, 003672, 003793, 001968, 003671) (Table 4). Other consortium members showed upregulation of some *ortho*-cleavage enzymes but lacked the full set required for conversion to 3-oxoadipate. In DEP1M, a protocatechuate 4,5-dioxygenase subunit *alpha/beta* was detected and upregulated on DEP compared to protocatechuate, but the complete enzymatic set for a *meta*-cleavage pathway was not detected.

**Table 4:** Presence of enzymes involved in the *ortho*-cleavage pathway of protocatechuate in the metaproteome of DEP grown cultures across all the strains of the microbial consortium.

Proteins detected in cultures grown on 2 mM DEP	DEP1C	DEP1M	DEP1T
Protocatechuate 3,4-dioxygenase subunit *alpha*			
Protocatechuate 3,4-dioxygenase subunit *beta*	**˄**	**˄**	
Carboxy-*cis,cis*-muconate cycloisomerase	**˄**		
Carboxymuconolactone decarboxylase	**˄**		
3-oxoadipate enol-lactonase	**˄**		

Green: Detected in the metaproteome when grown on 2 mM DEP. Letter up arrow head (˄) symbols indicate significant overproduction in cultures grown on DEP versus grown on succinate.

## 4 Discussion

### 4.1 Growth on DEP and substrate spectrum

The presence of two bacterial genera in the consortium growing on DEP, namely *Pseudomonas* (strains DEP1C and DEP1T) and *Microbacterium* (strain DEP1M), was confirmed through genomics and membrane fatty acids profiling. The observation that the consortium, but not individual isolates of DEP1T, can utilize 2 mM DEP as the sole carbon and energy source (Figure 1) supports the presence of a cooperative catabolic network. When the consortium was grown on protocatechuate, the reduced abundance of DEP1M indicated that this strain was either unable to efficiently utilize this substrate or was outcompeted by the *Pseudomonas* strains under these conditions. Together, these observations demonstrate the efficient degradation of DEP by the microbial consortium, accompanied by distinct intermediate formation (monoethyl phthalate) and substrate utilization patterns.

The growth yields for DEP decreased with increasing concentrations (Figure 2), reflecting the compound’s toxic effects. Exposure to organic solvents disrupts membrane integrity, triggering adaptive changes in membrane composition and activation of efflux systems. These protective responses require significant energy, diverting resources from biomass formation and resulting in lower overall yields (Neumann et al., 2005; Isken et al., 1999). The transient accumulation of monoethyl phthalate followed by its disappearance indicates that monoethyl phthalate serves as an intermediate in DEP degradation. The subsequent accumulation of phthalate at a stable concentration suggests that the consortium lacks the ability to further metabolize this compound when it is present in the supernatant (Figure 3). This observation was supported by growth experiments and UPLC analyses, both confirming that phthalate supplemented as substrate does not support growth (Table 2) probably due to the lack of uptake systems.

The ability of the consortium to grow on protocatechuate (3,4-dihydroxybenzoic acid) provides evidence for the presence of downstream aromatic degradation pathways. Finally, the consortium’s ability to grow on a variety of phthalate esters, including dimethyl, dipropyl, and dibutyl phthalate, demonstrates its metabolic versatility across substrates with different alkyl chain lengths (Table 2). This broad substrate range enhances the potential value of the consortium for biotechnological and environmental applications, as it can degrade multiple PAE commonly found as plasticizers in contaminated environments.

### 4.2 Initiation of DEP degradation and the formation of protocatechuate

Like other amphiphilic and hydrophobic compounds, PAEs can accumulate in the phospholipid bilayers of the bacterial cytoplasmic membrane. This interaction weakens the structural integrity of the membranes (Shariati et al., 2022; Beney and Gervais, 2001; Hazel and Williams, 1990; Heipieper and De Bont, 1994; Qiao et al., 2024). The outer membrane of Gram-negative bacteria allows passive transmembrane passage through protein channels (porins) for small hydrophilic molecules. Because of their amphiphilic structure, PAEs can permeate through the cell envelope and the cytoplasmic membrane to enter the cells. Direct permeation of PAEs through the cytomembrane is likely too harmful to be the primary uptake strategy in PAE-degrading strains. Instead, aromatic growth substrates are usually taken up by membrane proteins such as major facilitator superfamily (MFS) transporters (proton motive force dependent) and ATP-binding cassette (ABC) transporters (Hara et al., 2007; Hong et al., 2006; Dahyabhai et al., 2021; Rigel and Silhavy, 2012; Zampolli et al., 2019).

The biodegradation of PAEs typically starts with their hydrolysis into the corresponding monoalkyl ester, which is then hydrolyzed to phthalic acid, whereby the corresponding alkyl alcohols are formed. These reactions can be catalyzed by intra- or extracellular esterases (Niazi et al., 2001; Maruyama et al., 2005; Boll et al., 2020). Once one ester bond of the phthalate esters is cleaved, the singly charged monoesters can enter the cells by different mechanisms. Thus, for the degradation of DEP, it is likely that the first ester cleavage takes places extracellularly and that the resulting monoester can pass through the protein channels of the outer membrane and is subsequently taken up into the cells due to active transport or facilitated diffusion. Given the composition of the microbial consortium, which consists of two different genera, it is likely that the entire degradation pathway for DEP is not carried out by a single species, but rather that the tasks are distributed among the consortium members.

**Figure 5:**
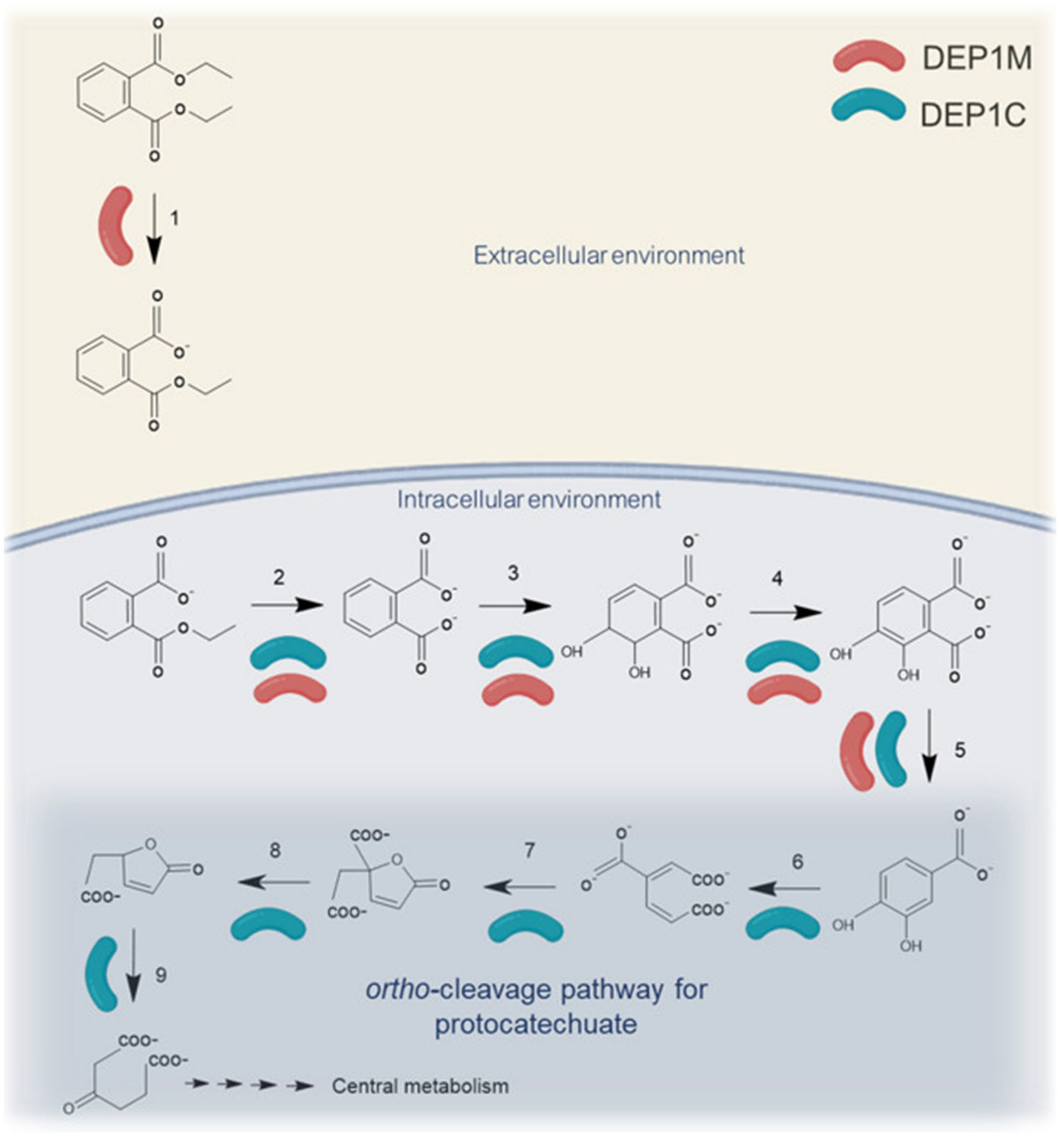
Illustration of the suggested degradation pathway for diethyl phthalate. DEP hydrolase is secreted by DEP1M. The degradation of monoethyl phthalate to protocatechuate can be carried by DEP1C and DEP1M. The ortho-cleavage pathway is most probably exclusively happing in DEP1C. (1) Diethyl phthalate hydrolase, (2) Monoethyl phthalate hydrolase, (3) Phthalate 3,4-dioxygenase, (4) Phthalate 3,4-cis-dihydrodiol dehydrogenase, (5) 3,4-dihydroxy-phthalate 2-decarboxylase, (6) 3,4-protocatechuate-dioxygenase, (7) 3-carboxymuconate cycloisomerase, (8) 4-carboxymuconolactone decarboxylase, (9) β-ketoadipate enol-lactone hydrolase. Metaproteomic analysis shows that the enzymes involved in step 1, 3, 4, 5, 6, 7, 8, 9 were upregulated on DEP when compared to Succinate. Enzymes involved in step 3, 4 and 5 were found in metaproteome but several candidates were identified.

The accumulation of phthalate in the supernatant most probably occurs by a side reaction of the DEP hydrolase of DEP1M (Table 3) cleaving the monoethyl phthalate to ethanol and phthalate, when the monoethyl phthalate is not taken up rapidly by the cells. The accumulation of phthalate in the supernatant of up to 0.2 mM and the fact that phthalate is not supporting growth can be explained by a lack of corresponding transporters. Phthalate transporters were documented for *Burkholderia* spp. and *Rhodococcus jostii* RHA1 (Chang et al., 2009; Hara et al., 2007; Hara et al., 2010). However, phthalate is an intermediate of PAE degradation (Boll et al., 2020) and also our suggested degradation pathway for DEP (Figure 5) contains an additional intracellular hydrolysis step leading to phthalate (numerous hydrolase candidates were identified in DEP-grown cells, data not shown). Subsequently, a phthalate dioxygenase dihydroxylates the aromatic ring forming a catecholic intermediate of phthalate (Nomura et al., 1992; Sasoh et al., 2006). DEP1C and DEP1M of the microbial consortium contain candidates for a phthalate 3,4-dioxygenase (Table 3). Notably, although the sequence similarity of pgaptmp_003809 from DEP1C is high enough to suggest that its molecular function is conserved, especially given the broad spectrum of substrates that dioxygenases can accept (Gibson and Parales, 2000), the differences in sequence indicate that this protein is an ortholog with some distinct features, and therefore requires further investigation (Joshi and Xu, 2007). The hydroxylated product is likely reduced and subsequently decarboxylated (Table 3). To sum up, the intracellular degradation of monoethyl phthalate leading to protocatechuate is likely to happen in both strains, DEP1C and DEP1M. However, the reduced abundance of DEP1M in cultures grown on protocatechuate suggests that this strain exhibits limited metabolic performance beyond this intermediate, indicating that the downstream catabolic steps are primarily carried out by DEP1C. Notably, in the case of protocatechuate, in contrast to phthalate, interspecies metabolite exchange appears to be possible.

*Pseudomonas* species have previously been documented as PAE degraders, isolated from environments such as wetlands, either as single strains or as members of microbial consortia (Shariati et al., 2021; Shariati et al., 2023). Another study reported a bacterial consortium in which *Microbacterium* sp. PAE-1 initiated DBP degradation through two sequential hydrolysis steps, while *Pandoraea* sp. PAE-2 completed the breakdown of phthalic acid. Within this consortium, *Microbacterium’s* novel esterases DpeH and MpeH played a crucial role, efficiently converting diverse dialkyl and monoalkyl phthalate esters into intermediates accessible for complete mineralization (Lu et al., 2020). Microbacterium strain DEP1M encodes homologs of DpeH and MpeH showing 97% (alpha/beta fold hydrolase pgaptmp_002293) and 99% (alpha/beta fold hydrolase pgaptmp_002294) sequence identity, respectively, and 100% query coverage. Both proteins are upregulated in DEP- versus succinate-grown cells. It is conceivable that pgaptmp_002293 cleaves the small amount of DEP that passively permeates through the cell wall and membrane, whereas pgaptmp_002294 represents an additional candidate responsible for further cleavage of the resulting monoester. The second hydrocarbonic product of the ester cleavage, ethanol, serves as a growth substrate for the microbial consortium.

### 4.3 Mineralization via the *ortho* cleavage pathway

Protocatechuate was shown to be a central degradation intermediate of PAEs and we could show that it was also degraded by our consortium (Boll et al., 2020). The cleavage of the aromatic ring of protocatechuate can occur either via the extradiolic (*meta*-cleavage) or intradiolic (*ortho*-cleavage) pathway (Harwood and Parales, 1996). In our consortium the complete *ortho*-cleavage pathway with protocatechuate 3,4-dioxygenase as ring cleaving enzyme was shown to be present and upregulated, especially in strain DEP1C (Table 4 and Supplementary Table S3). The other consortium members show upregulation of some *ortho*-cleavage enzymes but lack the complete set required for conversion to 3-oxoadipate under DEP-grown conditions. Thus, DEP1C seems to be the principal strain responsible for complete protocatechuate mineralization. DEP1C could outperform DEP1M and DEP1T in terms of uptake of the monoethyl phthalate as well as the formation and degradation of the central intermediate protocatechuate. This is supported by the dominance of strain DEP1C within the microbial consortium when grown on DEP and the underrepresentation of DEP1M when protocatechuate serves as a grown substrate (Figure 1) as well in the changing PLFA patterns (Supplementary Figure S1). However, the strain DEP1C as well as strain DEP1T are absolutely dependent on the extracellular cleavage of DEP to monoethyl phthalate. By enabling the substrate uptake, DEP1M plays a key role in the cross-feeding of the microbial community growing on DEP.

## 5 Conclusion

The research is relevant due to the environmental contamination caused by PAEs, which enter ecosystems primarily through the use of agricultural foils and accidental spills or leaks. The direct application of an immobilized microbial consortium to contaminated areas, known as bioaugmentation, might have the potential to accelerate the degradation of PAEs. This approach may also be effective in treating industrial plastic waste streams. Accelerated degradation of non-covalently bound PAEs can result in more brittle plastic, thereby increasing the surface area available for microbial attack and further enhancing the breakdown process.

## Data Availability

The datasets presented in this study can be found in online repositories. The metagenome derived genomes have been deposited to figshare, https://doi.org/10.6084/m9.figshare.31042420 and NCBI Bioproject PRJNA1333888, https://www.ncbi.nlm.nih.gov/bioproject/PRJNA1333888. The mass spectrometry proteomics data have been deposited to the ProteomeXchange Consortium via the PRIDE (Perez-Riverol et al., 2025) partner repository with the dataset identifier PXD073022 and 10.6019/PXD073022. R codes can be accessed under the following link: https://git.ufz.de/bertoldi/r_codes_publication.

## References

[ref1] Abueg L. A. L. Afgan E. Allart O. Awan A. Bacon W. (2024). The galaxy platform for accessible, reproducible, and collaborative data analyses: 2024 update. Nucleic Acids Res. 52, W83–W94. doi: 10.1093/nar/gkae410 38769056 PMC11223835

[ref2] Alneberg J. Bjarnason B. S. de Bruijn I. Schirmer M. Quick J. Ijaz U. Z. . (2014). Binning metagenomic contigs by coverage and composition. Nat. Methods 11, 1144–1146. doi: 10.1038/nmeth.3103 25218180

[ref3] Altschul S. F. Madden T. L. Schäffer A. A. Zhang J. Zhang Z. Miller W. . (1997). Gapped BLAST and PSI-BLASTT: a new generation of protein database search programs. NAR 25, 3389–3402. doi: 10.1093/nar/25.17.3389, 9254694 PMC146917

[ref4] Beney L. Gervais P. (2001). Influence of the fluidity of the membrane on the response of microorganisms to environmental stresses. Appl. Microbiol. Biotechnol. 57, 34–42. doi: 10.1007/s002530100754 11693931

[ref5] Benjamini Y. Hochberg Y. (1995). Controlling the false discovery rate: a practical and powerful approach to multiple testing. J. R. Stat. Soc. Ser. B Stat Methodol. 57, 289–300. doi: 10.1111/j.2517-6161.1995.tb02031.x

[ref6] Bligh E. G. Dyer W. J. (1959). A rapid method of total lipid extraction and purification. Can. J. Biochem. Physiol. 37:911–917. doi: 10.1139/o59-099 13671378

[ref7] Boll M. Geiger R. Junghare M. Schink B. (2020). Microbial degradation of phthalates: biochemistry and environmental implications. Environ. Microbiol. Rep. 12, 3–15. doi: 10.1111/1758-2229.12787 31364812

[ref8] Callahan B. J. McMurdie P. J. Rosen M. J. Han A. W. Johnson A. J. A. Holmes S. P. (2016). DADA2: high-resolution sample inference from Illumina amplicon data. Nat. Methods 13, 581–583. doi: 10.1038/nmeth.3869, 27214047 PMC4927377

[ref9] Carney Almroth B. Carmona E. Chukwuone N. Dey T. Slunge D. Backhaus T. . (2025). Addressing the toxic chemicals problem in plastics recycling. Camb. prisms Plast. 3:1–7. doi: 10.1017/plc.2025.1

[ref10] Chang H.-K. Dennis J. J. Zylstra G. J. (2009). Involvement of two transport systems and a specific porin in the uptake of phthalate by *Burkholderia* spp. J. Bacteriol. 191, 4671–4673. doi: 10.1128/JB.00377-09, 19429613 PMC2704722

[ref9001] Chaumeil P. A. Mussig A. J. Hugenholtz P. Parks D. H. (2022). GTDB-Tk v2: memory friendly classification with the genome taxonomy database. Bioinformatics 38, 5315–5316. doi: 10.1093/bioinformatics/btac672 36218463 PMC9710552

[ref9002] Chen Y. Chen Y. Shi C. Huang Z. Zhang Y. Li S. (2018). SOAPnuke: a MapReduce acceleration-supported software for integrated quality control and preprocessing of high-throughput sequencing data. Gigascience 7, 1–6. doi: 10.1093/gigascience/gix120 PMC5788068 29220494

[ref11] Dahyabhai P. J. Ulrich K. Mathias W. (2021). How to enter a bacterium: bacterial Porins and the permeation of antibiotics. Chem. Rev. 121, 5158–5192. doi: 10.1021/acs.chemrev.0c01213, 33724823

[ref9003] European Chemicals Agency. (2021). Guidance on Information Requirements and Chemical Safety Assessment. Helsinki: ECHA.

[ref13] European Human Biomonitoring Initiative (2025) Available online at: https://cordis.europa.eu/project/id/733032 (accessed October 2, 2025).

[ref14] Fauvelle V. Garel M. Tamburini C. Nerini D. Castro-Jiménez J. Schmidt N. . (2021). Organic additive release from plastic to seawater is lower under deep-sea conditions. Nat. Commun. 12:4426. doi: 10.1038/s41467-021-24738-w, 34285235 PMC8292457

[ref15] Frankenfield A. M. Ni J. Ahmed M. Hao L. (2022). Protein contaminants matter: building universal protein contaminant libraries for DDA and DIA proteomics. J. Proteome Res. 21, 2104–2113. doi: 10.1021/acs.jproteome.2c00145, 35793413 PMC10040255

[ref16] Frejno M. Berger M. T. Tüshaus J. Hogrebe A. Seefried F. Graber M. . (2025). Unifying the analysis of bottom-up proteomics data with CHIMERYS. Nat. Methods 22, 1017–1027. doi: 10.1038/s41592-025-02663-w, 40263583 PMC12074992

[ref17] Gibson D. T. Parales R. E. (2000). Aromatic hydrocarbon dioxygenases in environmental biotechnology. Curr. Opin. Biotechnol. 11, 236–243. doi: 10.1016/S0958-1669(00)00090-2, 10851146

[ref18] Girard L. Lood C. Höfte M. Vandamme P. Rokni-Zadeh H. van Noort V. . (2021). The ever-expanding *Pseudomonas* genus: description of 43 new species and partition of the *Pseudomonas putida* group. Microorganisms 9:1–41. doi: 10.3390/microorganisms9081766, 34442845 PMC8401041

[ref19] Gomila M. Peña A. Mulet M. Lalucat J. García-Valdés E. (2015). Phylogenomics and systematics in *Pseudomonas*. Front. Microbiol. 6:214. doi: 10.3389/fmicb.2015.00214, 26074881 PMC4447124

[ref20] Hara H. Eltis L. D. Davies J. E. Mohn W. W. (2007). Transcriptomic analysis reveals a bifurcated terephthalate degradation pathway in *Rhodococcus* sp. strain RHA1. J. Bacteriol. 189, 1641–1647. doi: 10.1128/JB.01322-06, 17142403 PMC1855752

[ref21] Hara H. Stewart G. R. Mohn W. W. (2010). Involvement of a novel ABC transporter and monoalkyl phthalate ester hydrolase in phthalate ester catabolism by *Rhodococcus jostii* RHA1. Appl. Environ. Microbiol. 76, 1516–1523. doi: 10.1128/AEM.02621-09, 20038686 PMC2832387

[ref22] Hartmans S. Smits J. P. Van Der Werf M. J. Volkering F. De Bont J. A. M. (1989). Metabolism of styrene oxide and 2-Phenylethanol in the styrene-degrading *Xanthobacter* strain 124X. Appl. Environ. Microbiol. 55, 2850–2855. doi: 10.1128/aem.55.11.2850-2855.1989, 16348047 PMC203180

[ref23] Harwood C. S. Parales R. E. (1996). The β-ketoadipate pathway and the biology of self-identity. Ann. Rev. Microbiol. 50, 553–590. doi: 10.1146/annurev.micro.50.1.553, 8905091

[ref24] Hazel J. R. Williams E. E. (1990). The role of alterations in membrane lipid composition in enabling physiological adaptation of organisms to their physical environment. Prog. Lipid Res. 29, 167–227. doi: 10.1016/0163-7827(90)90002-3, 2131463

[ref25] Heipieper H. De Bont J. A. M. (1994). Adaptation of *Pseudomonas putida* S12 to ethanol and toluene at the level of fatty acid composition of membranes. Am. Soc. Microbiol. 60:4440–4. doi: 10.1128/aem.60.12.4440-4444.1994 PMC202003 7811084

[ref26] Hesse C. Schulz F. Bull C. T. Shaffer B. T. Yan Q. Shapiro N. . (2018). Genome-based evolutionary history of *Pseudomonas* spp. Environ. Microbiol. 20, 2142–2159. doi: 10.1111/1462-2920.14130 29633519

[ref9004] Hofmann U. Schlosser D. (2016). Biochemical and physicochemical processes contributing to the removal of endocrine-disrupting chemicals and pharmaceuticals by the aquatic ascomycete Phoma sp. UHH 5-1-03. Appl Microbiol Biotechnol 100, 2381–2399. doi: 10.1007/s00253-015-7113-0 26536880

[ref27] Hong H. Patel D. R. Tamm L. K. van den Berg B. (2006). The outer membrane protein OmpW forms an eight-stranded beta-barrel with a hydrophobic channel. J. Biol. Chem. 281, 7568–7577. doi: 10.1074/jbc.M512365200, 16414958

[ref28] Isken Sonja Derks Antonie Wolffs Petra F. G. Antonie Bont Jan A. M. de (1999): Effect of organic solvents on the yield of solvent-tolerant *Pseudomonas putida* S12. In: Appl. Microbiol. Biotechnol. 65, 2631–2635. doi: 10.1128/aem.65.6.2631-2635.1999 PMC91388 10347053

[ref29] Joshi T. Xu D. (2007). Quantitative assessment of relationship between sequence similarity and function similarity. BMC Genomics 8:222. doi: 10.1186/1471-2164-8-222, 17620139 PMC1949826

[ref9005] Kang D. D. Li F. Kirton E. Thomas A. Egan R. An H. . (2019). MetaBAT 2: an adaptive binning algorithm for robust and efficient genome reconstruction from metagenome assemblies. PeerJ 7:e7359. doi: 10.7717/peerj.7359 31388474 PMC6662567

[ref30] Klaes S. Madan S. Deobald D. Cooper M. Adrian L. (2023). GroEL-Proteotyping of bacterial communities using tandem mass spectrometry. Int. J. Mol. Sci. 24:15692. doi: 10.3390/ijms242115692, 37958676 PMC10649880

[ref31] Kumari P. Bandyopadhyay S. Das S. K. (2013). *Microbacterium oryzae* sp. nov., an *actinobacterium* isolated from rice field soil. Int. J. Syst. Evol. Microbiol. 63, 2442–2449. doi: 10.1099/ijs.0.046870-0, 23203624

[ref32] Lange R. Vogel N. Schmidt P. Gerofke A. Luijten M. Bil W. . (2022). Cumulative risk assessment of five phthalates in European children and adolescents. Int. J. Hyg. Environ. Health 246:114052. doi: 10.1016/j.ijheh.2022.114052, 36323174

[ref9006] Li D. Liu C. M. Luo R. Sadakane K. Lam T. W. (2015). MEGAHIT: an ultra-fast single-node solution for large and complex metagenomics assembly via succinct de Bruijn graph. Bioinformatics 31, 1674–1676. doi: 10.1093/bioinformatics/btv033 25609793

[ref33] Lu M. Jiang W. Gao Q. Zhang M. Hong Q. (2020). Degradation of dibutyl phthalate (DBP) by a bacterial consortium and characterization of two novel esterases capable of hydrolyzing PAEs sequentially. Ecotoxicol. Environ. Saf. 195:110517. doi: 10.1016/j.ecoenv.2020.110517, 32220793

[ref34] Maruyama K. Akita K. Naitou C. Yoshida M. Kitamura T. (2005). Purification and characterization of an esterase hydrolyzing monoalkyl phthalates from *Micrococcus sp.* YGJ1. J. Biochem. 137, 27–32. doi: 10.1093/jb/mvi004, 15713880

[ref35] Monclús L. Arp H. P. H. Groh K. J. Faltynkova A. Løseth M. E. Muncke J. . (2025). Mapping the chemical complexity of plastics. Nature 643, 349–355. doi: 10.1038/s41586-025-09184-8, 40634741 PMC12240811

[ref36] Moreno J. Nielsen H. Winther O. Teufel F. (2024). Predicting the subcellular location of prokaryotic proteins with DeepLocPro. Bioinformatics 40:1–5. doi: 10.1093/bioinformatics/btae677, 39540738 PMC11645106

[ref37] Morrison W. R. Smith L. M. (1964). Preparation of fatty acid methyl esters and dimethylacetals from lipids with boron fluoride–methanol. J. Lipid Res. 5, 600–608. doi: 10.1016/S0022-2275(20)40190-7 14221106

[ref38] Neumann G. Cornelissen S. van Breukelen F. Hunger S. Lippold H. Loffhagen N. . (2006). Energetics and surface properties of *Pseudomonas putida* DOT-T1E in a two-phase fermentation system with 1-decanol as second phase. Appl. Environ. Microbiol. 72, 4232–4238. doi: 10.1128/AEM.02904-05, 16751536 PMC1489673

[ref39] Neumann G. Kabelitz N. Zehnsdorf A. Miltner A. Lippold H. Meyer D. . (2005). Prediction of the adaptability of *Pseudomonas putida* DOT-T1E to a second phase of a solvent for economically sound two-phase biotransformations. Appl. Environ. Microbiol. 71, 6606–6612. doi: 10.1128/AEM.71.11.6606-6612.2005, 16269688 PMC1287635

[ref40] Niazi J. H. Prasad D. T. Karegoudar T. B. (2001). Initial degradation of dimethylphthalate by esterases from *Bacillus* species. FEMS Microbiol. Lett. 196, 201–205. doi: 10.1111/j.1574-6968.2001.tb10565.x, 11267780

[ref41] Nomura Y. Nakagawa M. Ogawa N. Harashima S. Oshima Y. (1992). Genes in PHT plasmid encoding the initial degradation pathway of phthalate in *Pseudomonas putida*. J. Ferment. Bioeng. 74, 333–344. doi: 10.1016/0922-338X(92)90028-S

[ref9009] Nurk S. Meleshko D. Korobeynikov A. Pevzner P. A. (2017). MetaSPAdes: a new versatile metagenomic assembler. Genome Res. 27, 824–834. doi: 10.1101/gr.213959.116 28298430 PMC5411777

[ref9010] Olm M. R. Brown C. T. Brooks B. Banfield J. F. (2017). dRep: a tool for fast and accurate genomic comparisons that enables improved genome recovery from metagenomes through de-replication. ISME J. 11, 2864–2868. doi: 10.1101/gr.213959 28742071 PMC5702732

[ref9011] Parks D. H. Imelfort M. Skennerton C. T. Hugenholtz P. Tyson G. W. (2015). CheckM: assessing the quality of microbial genomes recovered from isolates, single cells, and metagenomes. Genome Res 25, 1043–1055. doi: 10.1101/gr.186072.114 25977477 PMC4484387

[ref9012] Parks D. H. Rinke C. Chuvochina M. Chaumeil P. A. Woodcroft B. J. Evans P. N. . (2017). Recovery of nearly 8,000 metagenome-assembled genomes substantially expands the tree of life. Nat Microbiol 2, 1533–1542. doi: 10.1038/s41564-017-0012-7 28894102

[ref9019] Perez-Riverol Y. Bandla C. Kundu D. J. Kamatchinathan S. Bai J. Hewapathirana S. . (2025). The PRIDE database at 20 years: 2025 update. Nucleic Acids Res. 53, D543–D553. doi: 10.1093/nar/gkae1011 39494541 PMC11701690

[ref42] Qiao P. Ying T. Gu M. Zhu J. Mei C. Hu T. . (2024). Assimilation of phthalate esters in bacteria. Appl. Microbiol. Biotechnol. 108:276. doi: 10.1007/s00253-024-13105-6, 38536521 PMC10973024

[ref43] Ren L. Lin Z. Liu H. Hu H. (2018). Bacteria-mediated phthalic acid esters degradation and related molecular mechanisms. Appl. Microbiol. Biotechnol. 102, 1085–1096. doi: 10.1007/s00253-017-8687-5, 29238874

[ref44] Rigel N. W. Silhavy T. J. (2012). Making a beta-barrel: assembly of outer membrane proteins in gram-negative bacteria. Curr. Opin. Microbiol. 15, 189–193. doi: 10.1016/j.mib.2011.12.007, 22221898 PMC3320693

[ref45] Saito T. Suzuki K. Yamamoto J. Fukui T. Miwa K. Tomita K. . (1989). Cloning, nucleotide sequence, and expression in *Escherichia coli* of the gene for poly(3-hydroxybutyrate) depolymerase from *Alcaligenes faecalis*. J. Bacteriol. 171, 184–189. doi: 10.1128/jb.171.1.184-189.1989, 2644188 PMC209571

[ref46] Sasoh M. Masai E. Ishibashi S. Hara H. Kamimura N. Miyauchi K. . (2006). Characterization of the terephthalate degradation genes of *Comamonas sp.* strain E6. Appl. Environ. Microbiol. 72, 1825–1832. doi: 10.1128/AEM.72.3.1825-1832.2006, 16517628 PMC1393238

[ref47] Schwengers O. Jelonek L. Dieckmann M. A. Beyvers S. Blom J. Goesmann A. (2021). Bakta: rapid and standardized annotation of bacterial genomes via alignment-free sequence identification. Microb. Genom. 7:1–13. doi: 10.1099/mgen.0.000685 PMC8743544 34739369

[ref48] Shariati S. Ebenau-Jehle C. Pourbabaee A. A. Alikhani H. A. Rodriguez-Franco M. Agne M. . (2022). Degradation of dibutyl phthalate by *Paenarthrobacter* sp. Shss isolated from Saravan landfill, Hyrcanian forests, Iran. Biodegradation 33, 59–70. doi: 10.1007/s10532-021-09966-7, 34751871 PMC8803807

[ref49] Shariati S. Pourbabaee A. A. Alikhani H. A. (2023). Biodegradation of diethyl phthalate and phthalic acid by a new indigenous *Pseudomonas putida*. Folia Microbiol. 68, 477–488. doi: 10.1007/s12223-022-01022-y, 36635520

[ref50] Shariati S. Pourbabaee A. A. Alikhani H. A. Rezaei K. A. (2021). Biodegradation of DEHP by a new native consortium An6 (*Gordonia* sp. and *Pseudomonas* sp.) adapted with phthalates, isolated from a natural strongly polluted wetland. Environ. Technol. Innov. 24:101936. doi: 10.1016/j.eti.2021.101936

[ref12] Souza TSPd Kawaguti HY (2021). Cellulases, hemicellulases, and pectinases: applications in the food and beverage industry Food Bioprocess Technol. 14 1446–1477 doi: 10.1007/s11947-021-02678-z

[ref51] Tanca A. Palomba A. (2024). Metaproteomic analysis of fecal samples from human subjects and rodent models. Methods Mol. Biol. 2820, 115–125. doi: 10.1007/978-1-0716-3910-8_11, 38941019

[ref52] Thompson R. C. Moore C. J. vom Saal F. S. Swan S. H. (2009). Plastics, the environment and human health: current consensus and future trends. Philos. Trans. R. Soc. Lond. Ser. B Biol. Sci. 364, 2153–2166. doi: 10.1098/rstb.2009.0053, 19528062 PMC2873021

[ref9007] Uritskiy G. V. DiRuggiero J. Taylor J. (2018). MetaWRAP-a flexible pipeline for genome-resolved metagenomic data analysis. Microbiome. 6:158. doi: 10.1186/s40168-018-0541-1 30219103 PMC6138922

[ref53] Wang M.-H. Chen C.-F. Albarico F. P. J. B. Chen C.-W. Dong C.-D. (2022). Occurrence and distribution of phthalate esters and microplastics in wastewater treatment plants in Taiwan and their toxicological risks. Chemosphere 307:135857. doi: 10.1016/j.chemosphere.2022.135857, 35940417

[ref54] Wiesinger H. Wang Z. Hellweg S. (2021). Deep dive into plastic monomers, additives, and processing aids. Environ. Sci. Technol. 55, 9339–9351. doi: 10.1021/acs.est.1c00976, 34154322

[ref55] Wright R. J. Bosch R. Gibson M. I. Christie-Oleza J. A. (2020). Plasticizer degradation by marine bacterial isolates: A Proteogenomic and Metabolomic characterization. Environ. Sci. Technol. 54, 2244–2256. doi: 10.1021/acs.est.9b05228, 31894974 PMC7031849

[ref9008] Wu Y. W. Simmons B. A. Singer S. W. (2016). MaxBin 2.0: an automated binning algorithm to recover genomes from multiple metagenomic datasets. Bioinformatics 32, 605–607. doi: 10.1093/bioinformatics/btv638 26515820

[ref56] Zampolli J. Zeaiter Z. Di Canito A. Di Gennaro P. (2019). Genome analysis and -omics approaches provide new insights into the biodegradation potential of *Rhodococcus*. Appl. Microbiol. Biotechnol. 103, 1069–1080. doi: 10.1007/s00253-018-9539-7 30554387

[ref57] Zheng L. Xiong Y. Wang R. Zhou P. Pan Y. Dong X. . (2024). Extraction of proteins from soil. Methods Mol. Biol. 2820, 29–39. doi: 10.1007/978-1-0716-3910-8_4, 38941012

[ref58] Zhu Q. Jia J. Zhang K. Zhang H. Liao C. (2019). Spatial distribution and mass loading of phthalate esters in wastewater treatment plants in China: an assessment of human exposure. Sci. Total. Env. 656, 862–869. doi: 10.1016/j.scitotenv.2018.11.458, 30625672

