# Supplementary material for: Cross-feeding drives degradation of phthalate ester plasticizers in a bacterial consortium
Source: Front Microbiol. 2026 Mar 18;16:1757196. doi: 10.3389/fmicb.2025.1757196 (PMC13038979; doi:10.3389/fmicb.2025.1757196)
Supplement: Supplementary file 1 [file Table_1.docx]

Supplementary Material

# Supplementary Figures and Tables

**Supplementary Table S1:** Putative DEP hydrolases identified in the metaproteomic analysis. The table lists NCBI-annotated protein names and accession numbers, predicted subcellular localization probabilities obtained using DeepLocPro 1.0, and the relative abundance of each protein under different growth conditions. Differential protein production was detected in the metaproteome of culture supernatants during growth on DEP compared to 6 h DEP or succinate controls. Red indicates upregulation, while blue indicates downregulation relative to the reference condition.

| **Strain** | **Accession**  **number** | **Protein** | **DeepLocPro 1.0**  **probability** | | | **Supernatant metaproteome** | | | | | | |
| --- | --- | --- | --- | --- | --- | --- | --- | --- | --- | --- | --- | --- |
|  |  |  |  |  |  | xh DEP vs 6h DEP | | | xh DEP vs  xh Succinate | | | |
|  |  |  | Extracellular | Outer membrane | Periplasmic | 24 | 30 | 48 | 0 | 24 | 30 | 48 |
| DEP1M | pgaptmp_001484 | Cellulase family glycosylhydrolase | 0.997 | 0 | 0 |  |  |  |  |  |  |  |
| DEP1M | \| pgaptmp_001431 \| \| --- \| | Extracellular catalytic domain type 1 short-chain-length polyhydroxyalkanoate depolymerase | 0.999 | 0 | 0 |  |  |  |  |  |  |  |
| DEP1C | \| pgaptmp_000170 \|  \| \| --- \| --- \| | Phospholipase | 0.417 | 0.050 | 0.378 |  |  |  |  |  |  |  |
| DEP1C | \| pgaptmp_000058 \|  \| \| --- \| --- \| | Acyloxyacyl hydrolase | 0 | 0.999 | 0 |  |  |  |  |  |  |  |

**Supplementary Table S2:** Putative phthalate dioxygenases and candidates for the following reduction (dehydrogenation) and decarboxylation step selected from best BLASTp alignments. Putative phthalate dioxygenase proteins were identified based on BLASTp alignments against 20 reference sequences from UniProt. Putative phthalate dehydrogenases proteins were identified based on BLASTp alignments against 4 reference sequences from UniProt. Putative dihydroxyphthalate decarboxylases proteins were identified based on BLASTp alignments against 5 reference sequences from UniProt. Selection criteria included ≥80% query coverage, ≥25% sequence identity, and E-value < 1×10⁻8. Differential protein production was detected in the metaproteome of cell pellets during growth on DEP compared to succinate or protocatechuate. Proteins showing significant differential abundance under distinct growth conditions are indicated on the right.

|  |  |  |  | |  |  |  | | | **Metaproteome** | | | | | | | |  |
| --- | --- | --- | --- | --- | --- | --- | --- | --- | --- | --- | --- | --- | --- | --- | --- | --- | --- | --- |
| **Strain** | **Accession number** | **Protein** | **Percentage identity (%)** | | **E-value** | **Query coverage (%)** | **Accession number UniProt - sequences from literature** | | | **xh DEP vs**  **xh Succinate** | | | | **xh DEP vs**  **xh Proto** | | | |  |
|  |  |  |  |  |  |  |  | | | 0 | 24 | 30 | 48 | 0 | 24 | 30 | 48 | |
| Hydroxylation | | | | | | | | | |  |  |  |  |  |  |  |  | |
| DEP1C | pgaptmp_003809 | anthranilate 1,2-dioxygenase large subunit | 33.1 | | 6.44E-65 | 94.9 | A0A2Z5UDX3 | | |  |  |  |  |  |  |  |  | |
| DEP1M | pgaptmp_002286 | aromatic ring-hydroxylating dioxygenase subunit alpha | 81.1 | | 0 | 85.1 | A0A1I4GN37 | | |  |  |  |  |  |  |  |  | |
| DEP1M | pgaptmp_002256 | Rieske 2Fe-2S domain-containing protein | 78.7 | | 0 | 94.7 | A0A1I4GN37 | | |  |  |  |  |  |  |  |  | |
| DEP1M | pgaptmp_002257 | 3-phenylpropionate/cinnamic acid dioxygenase subunit beta | 61.3 | | 2.68E-85 | 91.8 | Q0RWD5  (reviewed) | | |  |  |  |  |  |  |  |  | |
| Dehydrogenation | | | | | | | | | |  |  |  |  |  |  |  |  | |
| DEP1C | pgaptmp_001576 | 4-hydroxythreonine-4-phosphate dehydrogenase PdxA | | 32.209 | 6.14E-34 | 100 | | UPI00004E5877 (reviewed) |  |  |  |  |  |  |  |  |  | |
| DEP1C | pgaptmp_004318 | SDR family NAD(P)-dependent oxidoreductase | | 32.171 | 3.96E-21 | 94.8 | | Q68YA3 |  |  |  |  |  |  |  |  |  | |
| DEP1T | pgaptmp_003143 | 3-oxoacyl-ACP reductase family protein | | 31.818 | 4.21E-14 | 97.1 | | Q68YA3 |  |  |  |  |  |  |  |  |  | |
| DEP1T | pgaptmp_003177 | SDR family oxidoreductase | | 30.916 | 6.28E-15 | 96.3 | | Q68YA3 |  |  |  |  |  |  |  |  |  | |
| Decarboxylation | | | | | | | | | |  |  |  |  |  |  |  |  | |
| DEP1C | pgaptmp_001853 | class II aldolase/adducin family protein | | 26.126 | 3.62E-10 | 92.9 | | Q93UV7 (reviewed) |  |  |  |  |  |  |  |  |  | |
| DEP1M | pgaptmp_002253 | class II aldolase/adducin family protein | | 28.972 | 4.75E-12 | 89.5 | | Q93UV7 (reviewed) |  |  |  |  |  |  |  |  |  | |

**Supplementary Table S3:** Putative enzymes of the protocatechuate *ortho*-cleavage pathway in the metaproteomic analysis. Differential protein production was detected in the metaproteome of cell pellets during growth on DEP compared to 6 h DEP, succinate or protocatechuate. Red indicates upregulation, while blue indicates downregulation relative to the reference condition.

| **Strain** | **Protein** | **xh DEP vs**  **6h DEP** | | | **DEP vs**  **Succinate** | | | | **DEP vs Protocatechuate** | | | |
| --- | --- | --- | --- | --- | --- | --- | --- | --- | --- | --- | --- | --- |
|  |  | 24 | 30 | 48 | 0 | 24 | 30 | 48 | 0 | 24 | 30 | 48 |
| DEP1M | protocatechuate 3,4-dioxygenase subunit *alpha* |  |  |  |  |  |  |  |  |  |  |  |
|  | protocatechuate 3,4-dioxygenase subunit *beta* |  |  |  |  |  |  |  |  |  |  |  |
|  | carboxymuconolactone decarboxylase family protein |  |  |  |  |  |  |  |  |  |  |  |
| DEP1C | protocatechuate 3,4-dioxygenase subunit *alpha* |  |  |  |  |  |  |  |  |  |  |  |
|  | protocatechuate 3,4-dioxygenase subunit *beta* |  |  |  |  |  |  |  |  |  |  |  |
|  | muconate cycloisomerase family protein |  |  |  |  |  |  |  |  |  |  |  |
|  | 3-carboxy-*cis,cis-*muconate cycloisomerase |  |  |  |  |  |  |  |  |  |  |  |
|  | carboxymuconolactone decarboxylase family protein |  |  |  |  |  |  |  |  |  |  |  |
|  | carboxymuconolactone decarboxylase family protein |  |  |  |  |  |  |  |  |  |  |  |
|  | 3-oxoadipate enol-lactonase |  |  |  |  |  |  |  |  |  |  |  |
| DEP1T | protocatechuate 3,4-dioxygenase subunit *beta* |  |  |  |  |  |  |  |  |  |  |  |
|  | 3-carboxy-*cis,cis*-muconate cycloisomerase |  |  |  |  |  |  |  |  |  |  |  |
|  | 4-carboxymuconolactone decarboxylase |  |  |  |  |  |  |  |  |  |  |  |
|  | carboxymuconolactone decarboxylase family protein |  |  |  |  |  |  |  |  |  |  |  |
|  | 3-oxoadipate enol-lactonase |  |  |  |  |  |  |  |  |  |  |  |

**Supplementary Figure S1**: GC-FID analysis of the bacterial consortium on different carbon sources after 24 hours of growth. Average of three subsequent inoculations. Percentage of branched fatty acids are 20.3 ± 0.4% in 2 mM DEP, 30.7 ± 0.6% in 2 mM DEP + yeast extract, 1.5 ± 0.1% in 34 mM succinate, 3.6 ± 0.1% in 5 mM protocatechuate.

**Supplementary Table S4**: Table of Di-alkyl hydrolases known from Literature and their cellular localization probabilities.

| Protein name | Accession number | | Cellular localization  (DeepLocPro 1.0 probability) | |
| --- | --- | --- | --- | --- |
| DphBL1 | OM455495.1 | Extracellular | | 0.5752 |
| CarEW | KM098150.1 | Cytoplasmic | | 0.5141 |
| DpeH | MK165157.1 | Cytoplasmic | | 0.8035 |
| EstS | AEW03609.1 | Cytoplasmic | | 0.9831 |
| PehA | AAK16532.1 | Cytoplasmic | | 0.9768 |
| PatE | ABG99214.1 | Cytoplasmic | | 0.9787 |
| BaCEs04 | MK617184.1 | Cytoplasmic | | 0.9858 |
| GoEst15 | MH513611.1 | Extracellular | | 0.8288 |
| AB hydrolase | SPC10960.1 | Cytoplasmic | | 0.9471 |
| PE hydrolase | AFK31309.1 | Cytoplasmic | | 0.7078 |
| EstB | AJO67803.1 | Cytoplasmic | | 0.7628 |
| EstG | AJO67804.1 | Cytoplasmic | | 0.9488 |
| EstSP1 | WP_010186968.1 | Cytoplasmic | | 0.9963 |
| DphB | AGY55960.1 | Cytoplasmic | | 0.9927 |
| DphA | AGY55959.1 | Cytoplasmic | | 0.7078 |
